# Peripheral arterial tonometry cannot detect patients at low risk of coronary artery disease

**DOI:** 10.1007/s12471-015-0715-4

**Published:** 2015-05-29

**Authors:** M. van den Heuvel, O. Sorop, P.J. Musters, R.T. van Domburg, T.W. Galema, D.J. Duncker, W.J. van der Giessen, K. Nieman

**Affiliations:** 1 Department of Cardiology, Erasmus Medical Center Rotterdam, ‘s-Gravendijkwal 230, 3015 CE Rotterdam, The Netherlands; 2 Department of Radiology, Erasmus Medical Center Rotterdam, Utrecht, The Netherlands; 3 ICIN Netherlands Heart Institute, Utrecht, The Netherlands; 4 Department of Experimental Cardiology, Ee2355, Erasmus Medical Center, Dr. Molewaterplein 50–60, 3015 GE Rotterdam, The Netherlands

**Keywords:** Coronary artery disease, Peripheral vascular function, Noninvasive testing

## Abstract

**Background:**

Endothelial dysfunction precedes coronary artery disease (CAD) and can be measured by peripheral arterial tonometry (PAT). We examined the applicability of PAT to detect a low risk of CAD in a chest pain clinic.

**Methods:**

In 93 patients, PAT was performed resulting in reactive hyperaemia (RHI) and augmentation (AIx) indices. Patients were risk classified according to HeartScore, Diamond and Forrester pretest probability (DF), exercise testing (X-ECG), and computed tomography calcium scoring (CCS) and angiography (CTA). Correlations, risk group differences and prediction of revascularisation within 1 year were calculated.

**Results:**

RHI correlated with HeartScore (*r* = − 0.21, *p* = 0.05), AIx with DF (*r* = 0.26, *p* = 0.01). However, both were not significantly different between normal and ischaemic X-ECG groups. In addition RHI and AIx were similar between low risk as compared with intermediate-to-high risk, based on risk algorithms (RHI: 1.98 (0.67) vs 1.94 (0.78); AIx: 0.0 (21) vs 5.0 (25); *p* = NS), or CCS and CTA (RHI: 1.99 (0.58) vs 1.89 (0.82); AIx: − 2.0 (24) vs 4.0 (25); *p* = NS). Finally, RHI and AIx failed to predict revascularisation (RHI: OR 1.42, CI 0.65–3.1; AIx: OR 1.02, CI 0.98–1.05).

**Conclusions:**

PAT cannot detect a low risk of CAD, possibly because RHI and AIx versus X-ECG, CCS and CTA represent independent processes.

## Introduction

Chest pain is a common symptom that may be caused by obstructive coronary artery disease (CAD) and requires risk stratification to assess the probability of CAD [1]. Risk algorithms have been developed based on combinations of risk factors [2, 3]. However, these models tend to overestimate the prevalence of CAD [4], and are based on population calculations rather than a direct assessment of the atherosclerotic process within an individual. Diagnostic testing with exercise electrocardiography (X-ECG) is considered to be highly discriminative [5], however is often inconclusive. Alternative tests using pharmacological stress are usually not immediately available. Computed tomography (CT) is expanding in the workup [1], using coronary calcium scoring (CCS) [6] and computed tomographic angiography (CTA) [7]. However, there are associated disadvantages such as costs, radiation exposure and administration of contrast agent [8]. Therefore, a better discrimination of patients at low risk of clinically relevant CAD will facilitate more efficient use of subsequent diagnostics.

Endothelial dysfunction precedes and contributes to the atherosclerotic disease process. Peripheral arterial tonometry (PAT) has emerged as an easy, noninvasive test to study endothelial function at the fingertip [9, 10]. From one single PAT measurement both the reactive hyperaemia index (RHI), indicating endothelium-dependent vasodilation [11], and the augmentation index (AIx), indicating arterial stiffness [12], can be derived. RHI and AIx have shown to be altered in the presence of CAD [12–15], and can be used in CAD risk stratification [16]. However, although PAT appears promising in research settings, validation in the clinical setting beside conventional measures has only been limited [11]. Therefore, the objective of this cross-sectional study was to assess the applicability of PAT to detect low risk of clinically relevant CAD in unselected patients visiting a rapid-access outpatient chest pain clinic.

## Methods

### Study population

From September 2009 to February 2010, 93 consecutive patients with new onset stable chest pain without evidence of ongoing ischaemia and no prior history of CAD gave written consent to undergo finger plethysmography to measure PAT at the same time as their chest pain evaluation, after study approval by the Medical Ethics Committee. The sample size was comparable with previous PAT validation studies [11, 12, 16], and sufficiently large to observe discriminating tendencies [17]. There were no exclusion criteria. All patients were scheduled to undergo conventional diagnostics. Based on cut-off values of these diagnostics, patients at low risk of clinically relevant CAD were identified. This was verified by evaluating revascularised patients by percutaneous coronary intervention (PCI) or coronary artery bypass grafting (CABG) for up to 1 year.

### Risk profiling

Cardiovascular risk factors were summarised using the HeartScore risk algorithm [3] to assess the risk of 10-year mortality of CAD and the Diamond and Forrester model (DF) [2, 4] to assess the pretest probability of CAD.

### Exercise electrocardiography

X-ECG was performed by standardised protocol [5]. A non-diagnostic result was defined as discontinuation without evidence of myocardial ischaemia before reaching 85 % of the target heart rate. Results were described as being non-ischaemic, inconclusive or ischaemic. Only non-ischaemic and ischaemic groups were taken into comparative anal.

### Cardiac computed tomography

A non-enhanced CT scan was performed to assess the amount of coronary calcium, followed by a contrast-enhanced scan to assess plaque burden and presence of stenotic CAD. Image acquisition was conducted using a 128-slice dual-source CT (Siemens Flash, Forchheim, Germany). Coronary arteries were quantitatively evaluated per coronary segment for presence of atherosclerotic plaque and > 50 % stenosis [7]. CCS and total number of plaques and stenoses per patient were included in the analysis.

### Peripheral arterial tonometry

The PAT response was measured with the EndoPAT 2000 device (Itamar Medical Ltd., Caesarea, Israel), according to a standardised protocol allowing simultaneous determination of both RHI and AIx. An index of pulse wave amplitude at rest and during reactive hyperaemia resulted in RHI, a measure of endothelium-dependent vasodilation [9–11, 13, 16]. Augmentation of the central pulse pressure allowed determination of AIx, representing endothelium-dependent arterial stiffness [12, 18].

### Statistical analysis

Summary data are presented as numbers (proportion) or expressed as mean ± standard deviation or median (interquartile range). In case of non-normal distribution, data were logarithmically transformed before analysis. Correlations between RHI, AIx and conventional diagnostics were analysed using Pearson’s correlation coefficient. Differences between two groups were assessed by *t*-testing. The predictive value of RHI and AIx for revascularisation was analysed using logistic regression and described as odds ratio (OR) with corresponding 95 % confidence interval (CI). Two-tailed *p* < 0.05 was considered significant.

## Results

### Conventional diagnostic characteristics

Overall, patients were middle aged with equal distribution between genders (Table 1). None had a history of CAD, but cardiovascular risk factors were abundant and three quarters used cardiovascular medication. In all, conventional diagnostics besides PAT were performed to examine clinically relevant CAD (Table 2). This allowed us to examine correlations between PAT and conventional diagnostics, as well as sub-group analysis for these outcomes and prediction of revascularisation within 1 year.

**Table 1:** Patient characteristics

*Demographics*	
Age (years)	56 ± 11
Women	40 (43 %)
*Risk factors*	
Nicotine abuse	23 (25 %)
Hypertension	56 (60 %)
Diabetes mellitus	20 (22 %)
Dyslipidaemia	56 (60 %)
Body mass index (kg/m^2^)	28 ± 5
Family history of cardiovascular disease	44 (47 %)
History of vascular disease	9 (10 %)
Cardiovascular medication use	70 (75 %)
*Revascularisation*	
PCI	10 (11 %)
CABG	2 (2 %)

*PCI* percutaneous coronary intervention, *CABG* coronary artery bypass grafting.

**Table 2:** Diagnostic characteristics

*Risk scores*	
HeartScore low-intermediate risk < 5 %	50 (55 %)
HeartScore high risk ≥ 5 %	41 (45 %)
Median HeartScore	4 (6)
DF low pretest probability < 30 %	24 (26 %)
DF intermediate pretest probability 30–70 %	38 (41 %)
DF high pretest probability > 70 %	31 (33 %)
Median DF pretest probability	55 (51)
*X-ECG*	
X-ECG	85 (91 %)
Inconclusive	32 (38 %)
Non-ischaemic	44 (52 %)
Ischaemic	9 (11 %)
*CT*	
CCS	92 (99 %)
Median CCS	6.1 (94)
CTA	91 (98 %)
Median plaques	6.0 (14)
Median stenosis	0 (1)
*PAT*	
RHI	90 (97 %)
Median RHI	1.95 (0.76)
AIx	92 (99 %)
Median Aix	3.0 (23)

*DF* Diamond and Forrester model, *X-ECG* exercise electrocardiography, *CT* computed tomography, *CCS* coronary calcium scoring, *CTA* computed tomography angiography, *PAT* peripheral arterial tonometry, *RHI* reactive hyperaemia index, *AIx* augmentation index.

### Digital pulse arterial tonometry testing

**Fig. 1:**
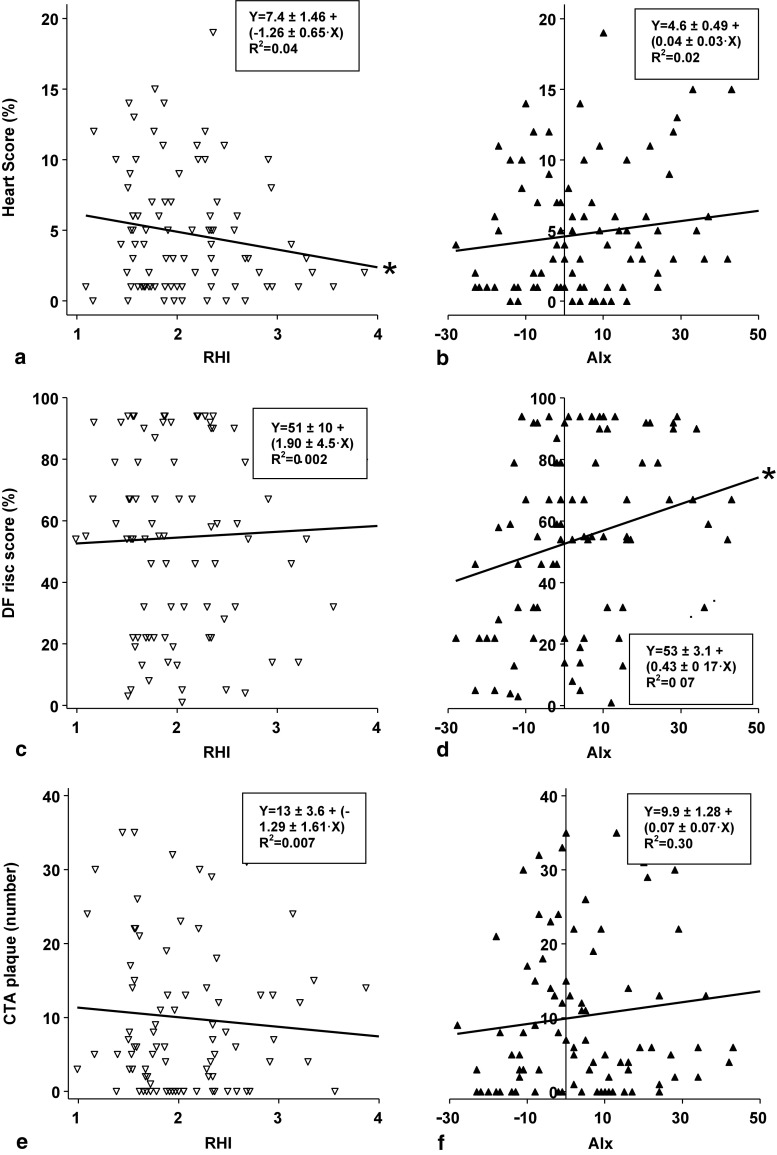
Correlation graphs of reactive hyperaemia index (RHI) (panels a, c, e) and augmentation index (AIx) (panels b, d, f) with HeartScore (panels a, b), Diamond and Forrester pretest probability (DF) (panels c, d) and total amount of plaque assessed by computed tomographic angiography (CTA) (panels e, f). Regression equation and R^2^ correlation coefficient are depicted per panel. Significant associations were observed between RHI and HeartScore as well as between AIx and DF, *P ≤ 0.05

With regard to risk algorithms, RHI correlated weakly with HeartScore (*r* = − 0.21, *p* = 0.05, Fig. 1a), whereas AIx did not (*r* = 0.15, *p* = NS, Fig. 1b). Surprisingly, RHI did not correlate with DF (*r* = 0.05, *p* = NS, Fig. 1c), whereas AIx did modestly (*r* = 0.26, *p* < 0.05, Fig. 1d). With regard to CT parameters, no significant correlations between RHI and CCS (*r* = − 0.09, *p* = NS), plaque (*r* = − 0.09, *p* = NS, Fig. 1e) or stenosis (*r* = − 0.08, *p* = NS) were found. Also no significant associations between AIx and CCS (*r* = 0.06, *p* = NS), plaque (*r* = 0.11, *p* = NS, Fig. 1f) or stenosis (*r* = 0.03, *p* = NS) were observed.

**Fig. 2:**
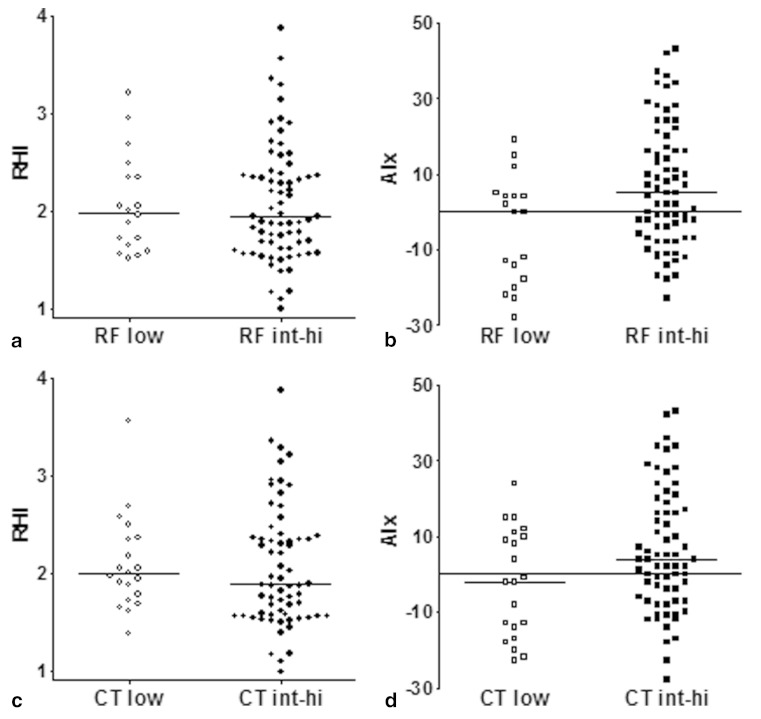
Differences of reactive hyperaemia index (RHI) (panels a, c) and augmentation index (AIx) (panels b, d) between patients at low and intermediate-to-high risk of clinically relevant CAD based on the combined outcome of risk scores (a, b), and CCS and CTA assessed plaque and stenosis (c, d). Horizontal bars depict the median value. No significant differences between the groups were observed. *RF* risk factors, *X-ECG* exercise ECG, *CT* computed tomography, *low* low risk, *int-hi* intermediate-to-high risk

To analyse low risk of clinically relevant CAD more specifically, patients were divided into low or intermediate-to-high risk groups based on the outcomes of risk scores, X-ECG and CT. According to the risk algorithms, patients at low risk (*n* = 18; defined as low risk of both DF and HeartScore outcomes) showed similar RHI and AIx values as compared with intermediate-to-high risk (*n* = 72; defined as intermediate-to-high risk of DF or HeartScore outcome), respectively (RHI: 1.98 (0.67) vs 1.94 (0.78); AIx: 0.00 (21) vs 5.0 (25); all *p* = NS; Fig. 2a, b). According to X-ECG results, low risk (*n* = 43; defined as a negative X-ECG outcome) also did not significantly differ from high risk (*n* = 7; defined as a positive outcome), respectively (RHI: 1.97 (0.72) vs 1.58 (0.61); AIx: − 0.50 (19) vs 8.0 (29); all *p* = NS). According to CT outcomes, low risk (*n* = 20; defined as absence of calcium, plaque and stenosis) resembled intermediate-to-high risk (*n* = 68; defined as presence of calcium, plaque or stenosis) (RHI: 1.99 (0.58) vs 1.89 (0.82); AIx: − 2.0 (24) vs 4.0 (25); all *p* = NS; Fig. 2c, d).

**Fig. 3:**
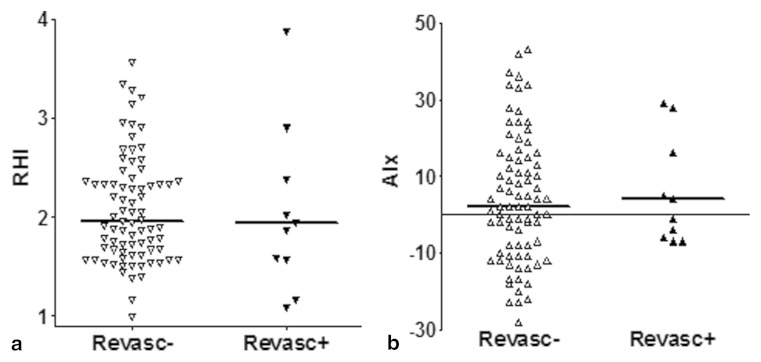
Differences of reactive hyperaemia index (RHI) (panel a) and augmentation index (AIx) (panel b) between patients with and without revascularisation up to 1 year after PAT measurement. Horizontal bars depict the median value. No significant differences between the groups were observed. *Revasc −* no revascularisation; *Revasc +* revascularisation

Finally, both RHI and AIx could not predict revascularisation within 1 year (RHI: OR 1.42, CI 0.65–3.1; AIx: OR 1.02, CI 0.98–1.05). Although RHI was lower in the revascularised group, this was not significantly different (revascularisation + (*n* = 11) vs revascularisation − (*n* = 79): 1.94 (1.06) vs 1.96 (0.70), *p* = NS; Fig. 3a). Also AIx showed a tendency towards an altered response in the revascularised group, however again not statistically significant (revascularisation + (*n* = 11) vs revascularisation − (*n* = 81): 4.0 (27) vs 2.0 (23), *p* = NS; Fig. 3b). This is in contrast to conventional diagnostics: the revascularised group (*n* = 7) showed significantly more ischaemic X-ECG outcomes than the non-revascularised group (*n* = 46) (0.71 ± 0.18 vs 0.09 ± 0.04; *p* < 0.01); CCS showed a modest prediction (OR 1.01, CI 1.00–1.03), whereas CTA-assessed plaque (OR 1.11, CI 1.05–1.17) and stenosis (OR 2.8, CI 1.60–4.8) showed strong predictions of revascularisation.

## Discussion

The present study examined the applicability of PAT-derived RHI and AIx to identify patients at low risk of clinically relevant CAD. The most important findings were (i) that although RHI and AIx correlated weakly with CAD risk estimates, (ii) both RHI and AIx were not related with X-ECG or CT-imaged parameters. (iii) Most importantly, low risk based on conventional diagnostics showed non-discriminative RHI and AIx outcomes. (iv) Finally, both RHI and AIx failed to predict revascularisation within 1 year.

### Clinical applicability of PAT

(i) We confirmed the modest relation of RHI with traditional risk factors, as reported previously [13]. AIx showed a weak correlation with the pretest probability of CAD. Patvardhan et al. [12] reported a strong association between AIx and age, most likely the basis for this correlation. (ii) No significant differences in either RHI or AIx were observed based on X-ECG outcome. This is remarkable since X-ECG is considered to be highly discriminative in patients at risk of obstructive CAD [5], and also RHI has shown to be able to discriminate between presence or absence of CAD [11]. Indeed, ischaemic X-ECG outcomes were more frequently present in revascularised patients of the present study whereas RHI was non-discriminative, making an independent relation likely. In addition, RHI and AIx did not significantly correlate with CCS, in contrast to a prior study of selected patients [19]. Han et al. [20] also showed no association between coronary endothelial dysfunction and calcification, reflecting that both can represent separate disease stages. In addition, brachial AIx was not independently associated with CCS [21], supporting our results. Indeed, other vascular function tests have previously indicated that endothelial dysfunction may represent an independent response in the progression of CAD, whereas a relation with the development of atherosclerosis exists [22–26]. RHI and AIx were not significantly associated with CTA-imaged plaque or stenosis. This is contrary to previous studies with imaged CAD describing specific subgroups with proven CAD [11, 12], diabetes [14], or women [15] although the presence of CAD was not verified with CTA in any of them. (iii) Also, RHI and AIx were not able to differentiate low risk of clinically relevant CAD, in contrast to results in selected populations [11, 12, 16], complicating comparison with our unselected population. (iv) Finally, RHI and AIx failed to predict revascularisation within 1 year, contrary to Rubinshtein, who showed that RHI independently predicted revascularisation [27]. Also radial AIx predicted revascularisation [28], although for digital AIx no such studies exist to our knowledge. However, again selected patients were evaluated with adverse events occurring at long-term follow-up, which is difficult to compare with our 1-year evaluation. Indeed, patient selection may strongly influence outcome because specific risk factors have additional effects on the measurement itself besides on the CAD process, as highlighted by the study of Gargiulo [14].

## Conclusions

In spite of the pathophysiological basis of endothelial dysfunction in CAD and the promising results with PAT in selected populations under controlled conditions, we found no evidence to support PAT as a diagnostic tool to detect low risk of clinically relevant CAD in an unselected outpatient population.

## References

[1] Montalescot G, Sechtem U, Achenbach S (2013). ESC guidelines on the management of stable coronary artery disease: the Task Force on the management of stable coronary artery disease of the European Society of Cardiology. Eur Heart J.

[2] Diamond GA (1983). A clinically relevant classification of chest discomfort. J Am Coll Cardiol.

[3] Thomsen T. (2005). HeartScore: a new web-based approach to European cardiovascular disease risk management. Eur J Cardiovasc Prev Rehabil.

[4] Genders TS, Steyerberg EW, Alkadhi H (2011). A clinical prediction rule for the diagnosis of coronary artery disease: validation, updating, and extension. Eur Heart J.

[5] Gibbons RJ, Balady GJ, Beasley JW (1997). ACC/AHA guidelines for exercise testing: executive summary. A report of the American College of Cardiology/American Heart Association Task Force on Practice Guidelines (Committee on Exercise Testing). Circulation.

[6] Dedic A, Rossi A, Ten Kate GJ (2013). First-line evaluation of coronary artery disease with coronary calcium scanning or exercise electrocardiography. Int J Cardiol.

[7] Nieman K, Galema T.W., Weustink A (2009). Computed tomography versus exercise electrocardiography in patients with stable chest complaints: real-world experiences from a fast-track chest pain clinic. Heart.

[8] Smith-Bindman R, Lipson J, Marcus R (2009). Radiation dose associated with common computed tomography examinations and the associated lifetime attributable risk of cancer. Arch Intern Med.

[9] Lekakis J, Abraham P, Balbarini A (2011). Methods for evaluating endothelial function: a position statement from the European Society of Cardiology Working Group on Peripheral Circulation. Eur J Cardiovasc Prev Rehabil.

[10] Flammer AJ, Anderson T, Celermajer DS (2012). The assessment of endothelial function: from research into clinical practice. Circulation.

[11] Kuvin JT, Mammen A, Mooney P (2007). Assessment of peripheral vascular endothelial function in the ambulatory setting. Vasc Med.

[12] Patvardhan E, Heffernan KS, Ruan J (2011). Augmentation index derived from peripheral arterial tonometry correlates with cardiovascular risk factors. Cardiol Res Pract.

[13] Hamburg NM, Keyes MJ, Larson MG (2008). Cross-sectional relations of digital vascular function to cardiovascular risk factors in the Framingham Heart Study. Circulation.

[14] Gargiulo P, Marciano C, Savarese G (2013). Endothelial dysfunction in type 2 diabetic patients with normal coronary arteries A digital reactive hyperemia study. Int J Cardiol.

[15] Matsuzawa Y, Sugiyama S, Sugamura K (2010). Digital assessment of endothelial function and ischemic heart disease in women. J Am Coll Cardiol.

[16] Heffernan KS, Karas RH, Patvardhan EA (2010). Peripheral arterial tonometry for risk stratification in men with coronary artery disease. Clin Cardiol.

[17] Sauder KA, West SG, McCrea CE (2014). Test-retest reliability of peripheral arterial tonometry in the metabolic syndrome. Diab Vasc Dis Res.

[18] Laurent S, Cockcroft J, Van Bortel L (2006). Expert consensus document on arterial stiffness: methodological issues and clinical applications. Eur Heart J.

[19] Li J, Flammer AJ, Nelson RE (2012). Normal vascular function as a prerequisite for the absence of coronary calcification in patients free of cardiovascular disease and diabetes. Circ J.

[20] Han SH, Gerber TC, Suwaidi JA (2010). Relationship between coronary endothelial function and coronary calcification in early atherosclerosis. Atherosclerosis.

[21] Park JS, Choi UJ, Lim HS (2011). The relationship between coronary artery calcification as assessed by multi-detector computed tomography and arterial stiffness. Clin Exp Hypertens.

[22] Jambrik Z, Venneri L, Varga A (2004). Peripheral vascular endothelial function testing for the diagnosis of coronary artery disease. Am Heart J.

[23] Frick M, Suessenbacher A, Alber HF (2005). Prognostic value of brachial artery endothelial function and wall thickness. J Am Coll Cardiol.

[24] Venneri L, Poggianti E, Jambrik Z (2007). The elusive prognostic value of systemic endothelial function in patients with chest pain syndrome. Int J Cardiol.

[25] Nishimura RA, Lerman A, Chesebro JH (1995). Epicardial vasomotor responses to acetylcholine are not predicted by coronary atherosclerosis as assessed by intracoronary ultrasound. J Am Coll Cardiol.

[26] Asselbergs FW, Monnink SH, Jessurun GA (2004). Assessing the prognostic value of coronary endothelial function in patients referred for a first coronary angiogram. Am J Cardiol.

[27] Rubinshtein R, Kuvin JT, Soffler M (2010). Assessment of endothelial function by non-invasive peripheral arterial tonometry predicts late cardiovascular adverse events. Eur Heart J.

[28] Weber T, Auer J, O’Rourke MF (2005). Increased arterial wave reflections predict severe cardiovascular events in patients undergoing percutaneous coronary interventions. Eur Heart J.

